# Association Between Iron Metabolism and Acute Kidney Injury in Critically Ill Patients With Diabetes

**DOI:** 10.3389/fendo.2022.892811

**Published:** 2022-04-29

**Authors:** Manqiu Mo, Yunqing Gao, Ling Deng, Yuzhen Liang, Ning Xia, Ling Pan

**Affiliations:** ^1^ Geriatric Department of Endocrinology, The First Affiliated Hospital of Guangxi Medical University, Nanning, China; ^2^ Department of Nephrology, The First Affiliated Hospital of Guangxi Medical University, Nanning, China; ^3^ Department of Endocrinology, The Second Affiliated Hospital of Guangxi Medical University, Nanning, China

**Keywords:** serum ferritin, diabetes mellitus, acute kidney injury, risk factors, survival

## Abstract

**Objective:**

Iron overload plays an important role in the pathogenesis of diabetes and acute kidney injury (AKI). The aim of this present study was to explore the relationship between iron metabolism and AKI in patients with diabetes.

**Methods:**

The clinical data of diabetes patients from MIMIC-III database in intensive care unit (ICU) were retrospectively analyzed. Regression analyses were used to explore the risk factors of AKI and all-cause death in critical patients with diabetes. Area under the receiver operating characteristic curves (AUROCs) were used to analyze serum ferritin (SF), and regression model to predict AKI in critical patients with diabetes. All diabetes patients were followed up for survival at 6 months, and Kaplan–Meier curves were used to compare the survival rate in patients with different SF levels.

**Results:**

A total of 4,997 diabetic patients in ICU were enrolled, with a male-to-female ratio of 1.37:1 and a mean age of 66.87 ± 12.74 years. There were 1,637 patients in the AKI group (32.8%) and 3,360 patients in the non-AKI group. Multivariate logistic regression showed that congestive heart failure (OR = 2.111, 95% CI = 1.320–3.376), serum creatinine (OR = 1.342, 95% CI = 1.192–1.512), Oxford Acute Severity of Illness Score (OR = 1.075, 95% CI = 1.045–1.106), increased SF (OR = 1.002, 95% CI = 1.001–1.003), and decreased transferrin (OR = 0.993, 95% CI = 0.989–0.998) were independent risk factors for AKI in critical patients with diabetes. Multivariate Cox regression showed that advanced age (OR = 1.031, 95% CI = 1.025–1.037), AKI (OR = 1.197, 95% CI = 1.011–1.417), increased Sequential Organ Failure Assessment score (OR = 1.055, 95% CI = 1.032–1.078), and increased SF (OR = 1.380, 95% CI = 1.038–1.835) were independent risk factors for 6-month all-cause death in critical diabetic patients. The AUROCs of SF and the regression model to predict AKI in critical patients with diabetes were 0.782 and 0.851, respectively. The Kaplan–Meier curve showed that the 6-month survival rate in SF-increased group was lower than that in SF-normal group (log-rank *χ* ^2^ = 16.989, *P* < 0.001).

**Conclusion:**

Critically ill diabetic patients with AKI were easily complicated with abnormal iron metabolism. Increase of SF is an important risk factor for AKI and all-cause death in critically ill patients with diabetes.

## Background

Acute kidney injury (AKI) is a sudden decline or loss of renal function. The number of hospital stays involving a principal diagnosis of AKI increased by a factor of 1.8 from 2005 to 2014, from 281,500 to 504,600 stays (1). About 50% of critically ill patients in intensive care unit (ICU) develop AKI (2, 3). In a multi-country prospective study, 11.0% of patients with severe AKI died in ICU, and AKI was associated with an increased risk of 28-day death (OR = 1.77, 95%CI = 1.17–2.68) (4). AKI is associated with an increase of ICU length of stay (LOS), use of renal replacement therapy (RRT), duration of mechanical ventilation, and mortality (5–8), which threatens human life and health and imposes a heavy burden on the global economy.

Diabetes, the most common cause of renal failure, is also associated with AKI and a higher risk of death from AKI (9, 10). Diabetes is one of the important risk factors for AKI (11). A study showed that patients with diabetes were eight times more likely to develop AKI than those without diabetes (12). AKI in diabetic patients is also an important cause of prolonged hospital stay, renal failure requiring RRT, development of chronic kidney disease (CKD) or end-stage renal disease, and eventual death (13, 14).

Iron is an essential trace element in the human body and plays an important role in many physiological and biochemical processes. When excess iron accumulates in organs such as the heart, kidney, and pancreas, the catalytic iron can produce toxic reactions leading to organ dysfunction (15). Iron overload has been linked to a higher risk of diabetes and insulin resistance (16, 17). In addition, ferroptosis plays an important role in the pathogenesis of AKI (18). Iron may increase the risk of kidney injury in diabetes by increasing oxidative/nitrification stress and decreasing antioxidant capacity (19, 20). However, the relationship between iron metabolism and AKI in diabetic patients remains unclear. The aims of this present study were to analyze the risk factors of AKI and all-cause death in critically ill patients with diabetes and to explore the association between iron metabolism and AKI in patients with diabetes.

## Materials and Methods

### The Data Source

We extracted data from the Medical Information Mart for Intensive Care III (MIMIC-III), a publicly available and freely available ICU database that records important information about over 50,000 adults admitted to the ICU at Beth Israel Deaconess Medical Center in Boston from 2001 to 2012 (21). The MIMIC-III database was approved by the Massachusetts Institute of Technology and the Institutional Review Boards. In order to obtain the permission of access to the database, we finished the National Institutes of Health’s web-based course, passed the Protecting Human Research Participants exam, and then obtained a certificate (number: 42064390).

### Inclusion and Exclusion Criteria

The inclusion criteria were as follows: (1) patients diagnosed with diabetes, (2) serum creatinine (Scr) levels were measured at least twice within 7 days, and (3) follow-up for at least 6 months after the diagnosis of AKI. The exclusion criteria were as follows: (1) age <18 years old, (2) stage 5 CKD or regular RRT, and (3) patients with missing important baseline clinical data.

### Data Extraction

We used structured query language (SQL) and PostgreSQL tools (version 9.6) to extract data from the MIMIC-III database. Data including age, sex, race, type of admission, vital signs, complications, scoring system, and laboratory parameters were collected. Complications include acute respiratory distress syndrome (ARDS), congestive heart failure (CHF), hypertension, diabetes, and CKD. All complications were collected based on international Classification of Diseases Version 9 codes recorded in MIMIC-III database. The scoring system includes the Simplified Acute Physiology Score II (SAPSII), Oxford Acute Severity of Illness Score (OASIS), and Sequential Organ Failure Assessment (SOFA) score. The laboratory parameters included white blood cell (WBC), hemoglobin (Hb), red blood cell (RBC), reticulocyte, platelet, random blood glucose (RBG), hemoglobin A1c (HbA1c), Scr, lactic acid, and iron metabolism indicators [serum iron, serum ferritin (SF), transferrin (TRF), total iron binding capacity (TIBC), and TRF saturation]. The scoring system data and laboratory parameters were obtained within 24 h of admission to the ICU. For patients with multiple admissions to the ICU, only data related to their first admission to the ICU were considered.

### Diagnostic Criteria

AKI was diagnosed in accordance with the diagnostic criteria in the guidelines of KDIGO: increase in Scr ≥26.5 μmol/L (0.3mg/dl) within 48 h or an increase from the baseline value by ≥50% within 7 days (22). Diabetes was defined as meeting the criteria of World Health Organization criteria [diabetic symptoms and (1) RBG ≥200 mg/dl or (2) fasting blood glucose ≥126 mg/dl or (3) postprandial blood glucose ≥200 mg/dl (23)] or a self-reported history of diabetes and/or receiving antidiabetic therapy at baseline.

### Research Methods and Grouping

According to the occurrence of AKI within 48 h after admission to ICU, the patients were divided into AKI group and non-AKI group. Because SF >1,000 ng/ml is regarded as iron overload, the patients were divided into SF-increased group (>1,000 ng/ml) and SF-normal group (≤1,000 ng/ml). All patients were followed up for the 6-month survival rate.

### Statistical Method

SPSS22.0 statistical software was used for statistical analysis. Measurement data consistent with normal distribution were expressed as mean ± standard deviation, and *T*-tests were used for comparison between groups. Measurement data of non-normal distribution were presented as M (1/4, 3/4), and Mann–Whitney *U*-tests were used for inter-group comparison. Count data were expressed as frequency (percentage), and *χ* ^2^ tests were used for comparison between groups. Multivariate logistic and Cox regression were used to explore the independent risk factors of AKI and all-cause death in critically ill patients with diabetes. Receiver operating characteristic curves (ROCs) were drawn to evaluate the prediction effect for AKI of SF values and regression model in critically ill patients with diabetes. The Kaplan–Meier curves (log-rank test) were used to compare the 6-month survival rate between AKI and non-AKI groups and in different SF levels. *P <*0.05 was considered statistically significant.

## Results

### Basic Characteristics of Patients

A total of 4,997 critically ill patients with diabetes were enrolled in this study, with a male-to-female ratio of 1.37:1, mean age of 66.87 ± 12.74 years, median hospital LOS of 13.5 (9.72–24.80) days, and median ICU LOS of 3.93 (2.77–6.85) days. Additionally, 1,178 patients (23.6%) had CKD, 3,529 patients (70.6%) had hypertension, 1,902 patients (38.1%) had CHF, and 1,087 patients (21.8%) had ARDS. All-cause mortality at 6-month was 47.6% (2378/4997), and hospital death occurred in 596 patients (11.9%). The incidence of AKI in critically ill patients with diabetes was 32.8% (1,637/4,997). There were 1,339 patients (81.8%) with stage 1 AKI, 136 patients (8.3%) with stage 2 AKI, and 162 patients (9.9%) with stage 3 AKI. The AKI etiologies were prerenal in 52.2%, renal in 35.3%, and postrenal in 12.5%.

### Comparison of Clinical Data Between the AKI Group and the Non-AKI Group

As shown in **Table 1**, the proportion of male, age, ICU LOS, the proportion of CHF, hypertension, CKD, ARDS, HbA1c, Scr, lactic acid, SOFA score, SAPSII, OASIS, SF, and TRF saturation in the AKI group were higher than those in the non-AKI group. Systolic blood pressure (SBP), diastolic blood pressure (DBP), mean arterial pressure (MAP), Hb, RBC, platelet, TRF, and TIBC were lower than those in the non-AKI group (*P* < 0.05). There were no significant differences in the type of diabetes, WBC, reticulocyte, RBG, and serum iron levels between the two groups (*P* > 0.05). In addition, the 6-month all-cause mortality was higher in the AKI group than that in the non-AKI group (53.8 *vs*. 44.6%, *P* < 0.001).

**Table 1:** Baseline characteristics between acute kidney injury (AKI) and non-AKI groups.

Parameters	AKI	Non-AKI	*t*/*Z*/*χ* ^2^	*P*-value
Male/female	985/652	1,903/1,457	5.636	0.018
Age (year)	67.48 ± 11.91	66.58 ± 13.11	2.430	0.015
Type of diabetes			2.533	0.282
Type 1	170 (10.4)	329 (9.8)		
Type 2	1,395 (85.2)	2,850 (84.8)		
Other types	72 (4.4)	181 (5.4)		
ICU LOS (day)	4.26 (2.98, 7.96)	3.72 (2.68, 6.27)	-7.485	<0.001
Ventilation, *n* (%)	1,260 (77.0)	2,121 (63.1)	96.428	<0.001
CHF, *n* (%)	738 (45.1)	1,164 (34.6)	50.886	<0.001
Hypertension, *n* (%)	1,235 (75.4)	2,294 (68.3)	27.268	<0.001
CKD, *n* (%)	634 (38.7)	544 (16.2)	310.362	<0.001
ARDS, *n* (%)	402 (24.6)	685 (20.4)	11.246	0.001
Height (cm)	169.09 ± 10.72	169.87 ± 12.50	-1.551	0.121
Weight (kg)	89.07 ± 24.43	88.62 ± 26.83	0.549	0.583
BMI (kg/m^2^)	31.06 ± 7.57	30.92 ± 8.48	0.417	0.676
SBP (mmHg)	122.47 ± 25.06	125.36 ± 24.19	-3.907	<0.001
DBP (mmHg)	60.93 ± 14.90	63.24 ± 15.08	-5.067	<0.001
PP (mmHg)	61.24 ± 21.12	61.87 ± 20.21	-1.019	0.308
MAP (mmHg)	80.82 ± 17.61	83.52 ± 17.11	-5.179	<0.001
WBC (×10^9^/L)	11.68 ± 6.35	12.21 ± 10.38	-1.895	0.058
Hb mg/dl	11.30 ± 2.11	11.61 ± 2.19	-4.800	<0.001
RBC (×10^12^/L)	3.91 ± 0.72	3.99 ± 0.73	-3.772	<0.001
Reticulocyte (%)	2.44 ± 1.72	2.29 ± 1.34	1.469	0.142
Platelet (×10^9^/L)	231.28 ± 108.73	248.41 ± 116.25	-4.967	<0.001
RBG (mg/dl)	200.64 ± 122.11	193.21 ± 115.52	1.880	0.060
HbA1c (%)	7.45 ± 1.87	7.26 ± 1.65	2.351	0.019
Scr (mg/dl)	2.88 ± 2.27	1.43 ± 1.23	24.051	<0.001
Lactic acid (mmol/L)	2.43 ± 2.05	2.20 ± 1.67	3.469	0.001
SOFA score	6.49 ± 3.34	4.25 ± 2.78	23.377	<0.001
SAPSII	43.99 ± 14.51	36.21 ± 12.45	18.628	<0.001
OASIS	36.13 ± 9.19	32.44 ± 8.39	13.691	<0.001
Serum iron (μmol/L)	45.97 ± 33.40	47.65 ± 38.93	-0.923	0.356
TRF (ng/ml)	165.70 ± 61.15	182.97 ± 65.67	-5.387	<0.001
TIBC (μmol/L)	215.47 ± 79.48	237.91 ± 85.38	-5.382	<0.001
SF (μg/L)	552.50 (305.50, 892.75)	215.00 (88.00, 450.00)	-17.271	<0.001
TRF saturation	0.24 ± 0.21	0.22 ± 0.18	2.295	0.022
Hospital death, *n* (%)	295 (18.0)	301 (9.0)	86.085	<0.001
6-month death, *n* (%)	880 (53.8)	1,498 (44.6)	37.250	<0.001

ARDS, acute respiratory distress syndrome; BMI, body mass index; CHF, congestive heart failure; CKD, chronic kidney disease; DBP, diastolic blood pressure; Hb, hemoglobin; HbA1c, hemoglobin A1c; ICU LOS, intensive care unit length of stay; MAP, mean arterial pressure; OASIS, oxford acute severity of illness score; PP, pulse pressure; RBC, red blood cell; SAPSII, simplified acute physiology score II; SBP, systolic blood pressure; Scr, serum creatinine; SF, serum ferritin, SOFA, sequential organ failure assessment; TIBC, total iron binding capacity; TRF, transferrin; WBC, white blood cell.

### Risk Factors for AKI in Critically Ill Patients With Diabetes

As shown in **Table 2**, the univariate logistic regression analysis showed the male sex, age, ICU LOS, ventilation, CHF, HBP, CKD, ARDS, SBP, DBP, MAP, Hb, RBC, platelet, HbA1c, Scr, lactic acid, SOFA score, SAPSII, OASIS, TRF, TIBC, SF, and TRF saturation were risk factors for AKI in critically ill patients with diabetes (*P* < 0.05). The above-mentioned variables were substituted into multivariate logistic regression, and the results showed that CHF (OR = 2.111, 95% CI = 1.320–3.376), increased Scr (OR = 1.342, 95% CI = 1.192–1.512), increased OASIS (OR = 1.075, 95% CI = 1.045–1.106), increased SF (OR = 1.002, 95% CI = 1.001–1.003), and decreased TRF (OR = 0.993, 95% CI = 0.989–0.998) were independent risk factors for AKI in critical diabetic patients.

**Table 2:** Logistic regression analysis for acute kidney injury in critically ill patients with diabetes.

Parameters	Univariate analysis			Multivariate analysis		
	OR	95%CI	*P*-value	OR	95%CI	*P*-value
Male	1.157	1.026–1.304	0.018			
Age	1.006	1.001–1.010	0.019			
ICU LOS	1.027	1.019–1.035	<0.001			
Ventilation	1.985	1.733–2.274	<0.001			
CHF	1.549	1.373–1.747	<0.001	2.111	1.320–3.376	0.002
HBP	1.428	1.249–1.632	<0.001			
CKD	3.272	2.858–3.746	<0.001			
ARDS	1.271	1.105–1.463	0.001			
SBP	0.995	0.993–0.998	<0.001			
DBP	0.990	0.986–0.994	<0.001			
MAP	0.991	0.988–0.994	<0.001			
Hb	0.936	0.911–0.962	<0.001			
RBC	0.856	0.789–0.928	<0.001			
Platelet	0.999	0.998–0.999	<0.001			
HbA1c	0.941	0.893–0.992	0.024			
Scr	1.798	1.708–1.892	<0.001	1.342	1.192–1.512	<0.001
Lactic acid	1.068	1.031–1.106	<0.001			
SOFA score	1.266	1.239–1.293	<0.001			
SAPSII	1.044	1.039–1.049	<0.001			
OASIS	1.050	1.042–1.057	<0.001	1.075	1.045–1.106	<0.001
TRF	0.996	0.994–0.997	<0.001	0.993	0.989–0.998	0.008
TIBC	0.997	0.995–0.998	<0.001			
SF	1.003	1.003–1.004	<0.001	1.002	1.001–1.003	<0.001
TRF saturation	1.793	1.086–2.960	0.023			

ARDS, acute respiratory distress syndrome; CHF, congestive heart failure; CKD, chronic kidney disease; DBP, diastolic blood pressure; Hb, hemoglobin; HbA1c, hemoglobin A1c; MAP, mean arterial pressure; OASIS, oxford acute severity of illness score; RBC, red blood cell; SAPSII, simplified acute physiology score II; SBP, systolic blood pressure; Scr, serum creatinine; SF, serum ferritin, SOFA, sequential organ failure assessment; TIBC, total iron binding capacity; TRF, transferrin.

### Risk Factors for All-Cause Death in Critically Ill Patients With Diabetes

As shown in **Table 3**, the univariate Cox regression analysis showed that male sex, CHF, AKI, ARDS, MAP, SOFA score, SAPSII, OASIS, TRF, TIBC, SF, and TRF saturation were risk factors for all-cause death in critically ill patients with diabetes (*P* < 0.05). The above-mentioned variables were substituted into multivariate Cox regression, and the results showed that advanced age (OR = 1.031, 95% CI = 1.025–1.037), AKI (OR = 1.197, 95% CI = 1.011–1.417), increased SOFA score (OR = 1.055, 95% CI = 1.032–1.078), and increased SF (OR = 1.380, 95% CI = 1.038–1.835) were independent risk factors for 6-month all-cause death in critical diabetic patients (*P* < 0.05).

**Table 3:** Cox regression analysis for all-cause death in critically ill patients with diabetes.

Parameters	Univariate analysis			Multivariate analysis		
	OR	95%CI	*P*-value	OR	95%CI	*P*-value
Age	1.032	1.028–1.036	<0.001	1.031	1.025–1.037	<0.001
CHF	1.397	1.288–1.515	<0.001			
AKI	1.341	1.234–1.457	<0.001	1.197	1.011–1.417	0.036
ARDS	1.715	1.567–1.878	<0.001			
MAP	0.994	0.992–0.997	<0.001			
WBC	1.008	1.005–1.011	<0.001			
SOFA score	1.075	1.062–1.089	<0.001	1.055	1.032–1.078	<0.001
SAPSII	1.030	1.027–1.032	<0.001			
OASIS	1.036	1.031–1.041	<0.001			
TRF	0.996	0.995–0.997	<0.001			
TIBC	0.997	0.996–0.998	<0.001			
Increased SF	1.708	1.320–2.210	<0.001	1.380	1.038–1.835	0.027
TRF saturation	2.271	1.662–3.104	<0.001			

ARDS, acute respiratory distress syndrome; CHF, congestive heart failure; MAP, mean arterial pressure; OASIS, oxford acute severity of illness score; SAPSII, simplified acute physiology score II; SF, serum ferritin, SOFA, sequential organ failure assessment; TIBC, total iron binding capacity; TRF, transferrin.

### Regression Model Equation and ROC Curve for Predicting AKI

**Figure 1:**
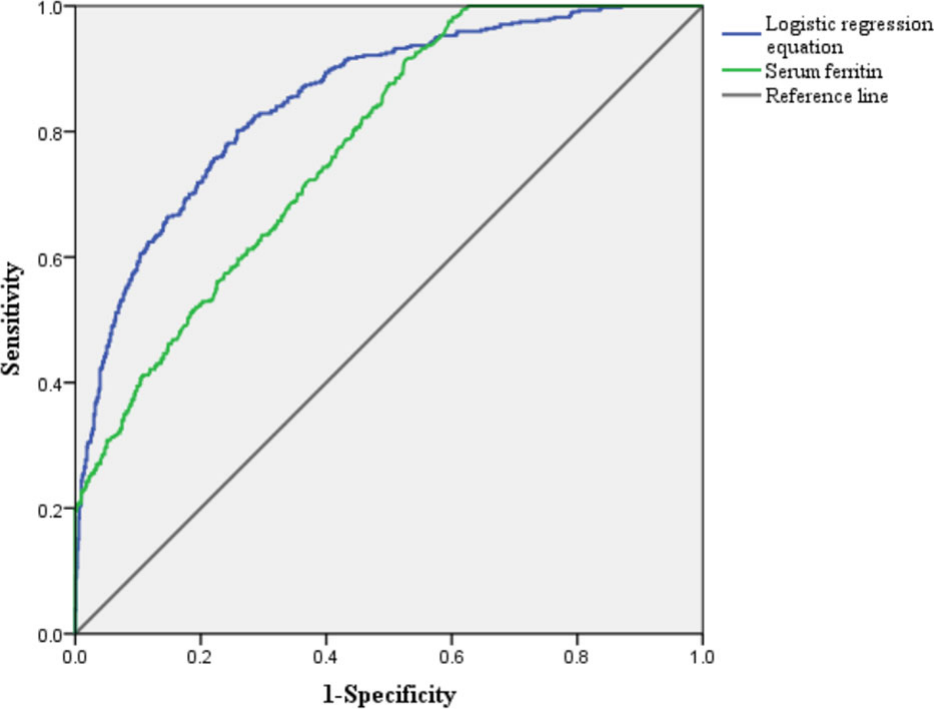
Receiver operating characteristic curve for predicting acute kidney injury in critically ill patients with diabetes.

A binary logistic regression equation was constructed as follows: *P* = 1/(1 + *e* ^–y^), *y* = –4.054 + 0.747X_1_ + 0.294X_2_ +0.073X_3_ - 0.007X_4_ + 0.002X_5_ (X_1_: CHF, X_2_: Scr, X_3_: OASIS, X_4_: SF, X_5_: TRF). The area under ROC (AUROC) of regression equation to predict AKI was 0.851, and the sensitivity, specificity, and Yoden index were 0.802, 0.741, and 0.543, respectively. The AUROC of SF to predict AKI in critical diabetic patients was 0.782, with a threshold of 204.5 ng/ml. The sensitivity, specificity, and Yoden index were 0.915, 0.615, and 0.500, respectively (**Figure 1**).

### Differences in 6-Month Survival Rate Among Different Groups

**Figure 2:**
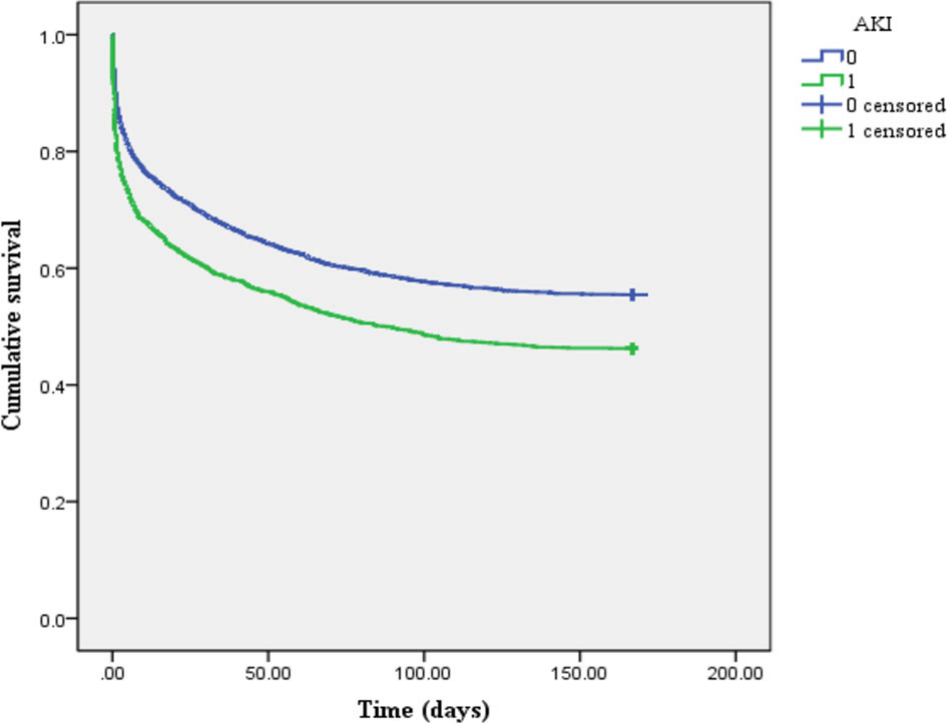
Kaplan–Meier survival curve of patients between acute kidney injury (AKI) and non-AKI groups (1 represents AKI group, and 0 represents non-AKI group).

**Figure 3:**
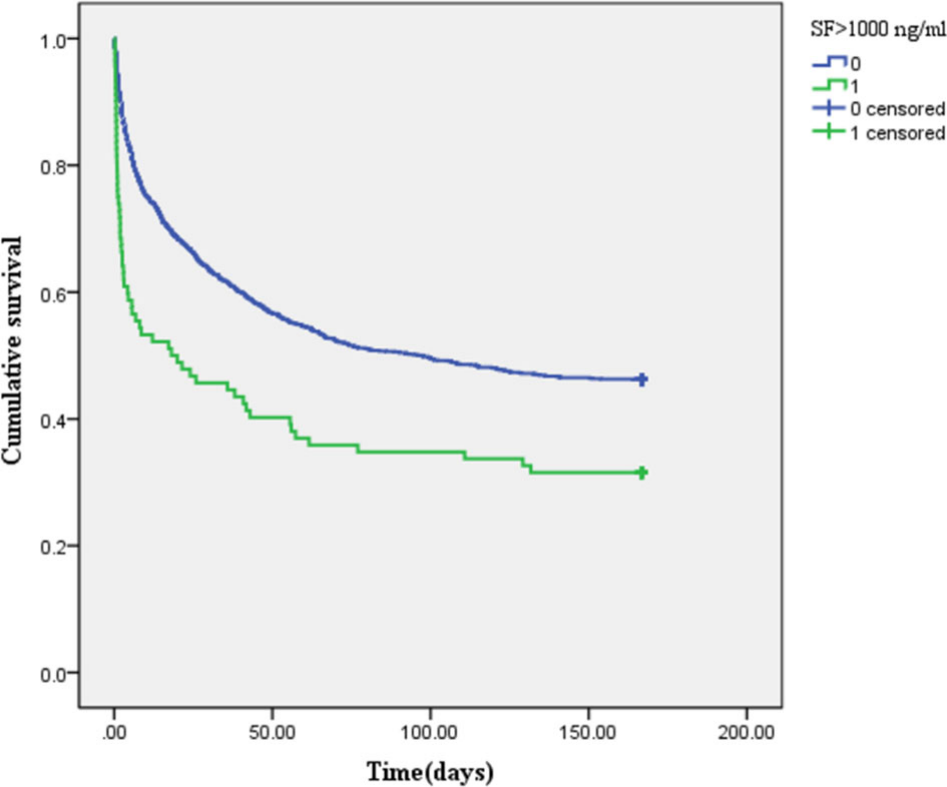
Kaplan–Meier survival curve of patients in the different serum ferritin (SF)-level groups (1 represents SF-increased group: >1,000 ng/ml, and 0 represents SF-normal group: ≤1,000 ng/ml).

The Kaplan–Meier survival curve (**Figure 2**) showed that the mean survival time of patients with AKI was less than that of non-AKI (88.92 *vs*. 107.25 days), and the 6-month cumulative survival rate of critical diabetic patients with AKI was lower than that of non-AKI (log-rank *χ* ^2^ = 47.962, *P* < 0.001). The mean survival time of patients in the SF-increased group (>1,000 ng/ml) was less than that in the SF-normal group (≤1,000 ng/ml) (65.53 *vs*. 136.85 days), and the 6-month cumulative survival rate of the SF-increased group was significantly lower than that of the SF-normal group (log-rank *χ* ^2^ = 16.989, *P* < 0.001; as shown in **Figure 3**).

## Discussion

In this study, the clinical data from MIMIC-III database were retrospectively analyzed, and the results showed that the critically ill patients with diabetes complicated with AKI were more likely to have abnormal iron metabolism indicators than those in non-AKI group, and the increase of SF was closely related to AKI and all-cause death in critical patients with diabetes. Previous studies have shown that the predictors for AKI in diabetic patients were advanced age, CHF, CKD, hypertension, smoking, alcohol consumption, diabetic nephropathy, infection, aminoglycosides, diabetic ketoacidosis, non-steroidal anti-inflammatory drugs, long course of diabetes, diuretics, beta blockers, and delay of follow-up (14, 24–26). However, whether abnormal iron metabolism is related to the occurrence of AKI in diabetic patients seems to be unproven in previous studies. This present study, for the first time, confirmed that increase of SF is an important risk factor and predictor of all-cause death and AKI, so as to provide certain reference for the prevention and treatment of AKI in critical patients with diabetes. Early correction of the iron metabolism disorder may improve the renal prognosis and survival rate of critical patients with diabetes.

Our results showed that critical diabetic patients with AKI were prone to increased SF and TRF saturation and decreased TRF and TIBC. Iron has many biological functions, such as regulating cell cycle progression, DNA synthesis and repair, mitochondrial function, and inflammatory responses (27). SF is an acute-phase reactant and can be elevated in a wide range of conditions, including acute liver injury, chronic liver disease, sepsis, systemic inflammatory conditions, inherited haemoglobinopathies, and secondary iron overload syndrome (28). Therefore, iron metabolism disorder and acute inflammatory response can both cause the increase of SF levels. In addition, our study showed that the increase of SF was an independent risk factor for AKI and 6-month all-cause death in critically ill patients with diabetes (the ORs were 1.002 and 1.380, respectively). Studies have also shown that increased SF was associated with decreased residual renal function and increased mortality in patients on peritoneal dialysis (29, 30), which were consistent with our results. The high glucose toxicity in diabetic patients could reduce the release of serum iron and increase the levels of SF. Iron overload can catalyze the production of excess hydroxyl free radicals and promote lipid peroxidation, resulting in oxidative stress and tissue damage and promoting the occurrence of AKI (31). Ferroptosis is also a type of cell death characterized by intracellular iron overload and the accumulation of reactive oxygen species, leading to lipid peroxidation, which is involved in the occurrence and development of AKI (18, 32, 33). In addition, excessive iron deposition in the liver, heart, pancreas, brain, skin, and other organs and tissues will not only lead to extensive fibrosis and impaired function of organs but also seriously affect the quality of life of patients and even lead to death (34). Therefore, diabetic patients with increased SF were more likely to develop AKI and have a high risk of all-cause death. However, there was also a study that did not match our results. In a prospective cohort study, SF levels at admission could be used as a prognostic indicator of renal recovery in patients with AKI, in which SF alleviated oxidative stress by sequestration of iron and limited its involvement in chemical reactions that produce reactive oxygen species, thus helping to improve the renal prognosis (35). The possible reasons were the difference in basic characteristics, basic diseases, and therapeutic measures for the renal protection of patients. The TRF is the main iron-containing protein in plasma, which is responsible for the transport of iron released by erythrocyte degradation and absorbed by the digestive tract. When TRF decreases, the catalytic transport of iron will be inhibited. In addition, the concentration of TRF will also decrease in inflammatory and malignant lesions, which further affects the occurrence of renal inflammation (36). Combined with the results of our study, the increased SF in critically ill diabetic patients with AKI might be caused by inflammation and iron metabolism disorder at the same time. However, which kinds of mechanism play a dominant role in the pathogenesis of AKI in critically ill diabetic patients still need to be further studied.

In addition, our study also showed that CHF was an independent risk factor for diabetic AKI. In a retrospective study with a median follow-up time of 30.75 months, 19.76% of patients with type 2 diabetes had AKI, and CHF (adjusted OR = 2.89, 95%CI = 1.62–5.13) was an important predictor of AKI (37). CHF is often associated with the state of low cardiac output resulting in renal hypoperfusion. Morever, blocked venous return also leads to ischemic renal injury, which is also referred to as acute tubular necrosis (38). Ultimately, decreased renal blood flow leads to epithelial and necrotic fragmentation obstruction and loss of filtration fluid through damaged tubular epithelium, and decreased glomerular ultrafiltration pressure leads to decreased glomerular filtration rate and increased Scr (39, 40). In addition, we also included in the regression model of the OASIS the Scr levels as predictors of AKI in diabetes. A study showed that the OASIS had a good predictive effect on the assessment and prognosis of critically ill patients (41). The baseline Scr values could also predict the occurrence and progression of AKI (42). These results were all consistent with our findings.

However, several limitations to our study should be acknowledged. First, our study was a single-center retrospective analysis, and the subjects were all critically ill patients in ICU. Second, the diagnosis of AKI was determined only by changes in Scr; some AKI patients with only changes in urine volume might be missed. Third, due to data limitations, only the results of iron metabolism laboratory parameters were collected in this study, without collecting the results of iron deposition in organs (such as through magnetic resonance imaging). Fourth, the study did not include levels of inflammation indicators (C-reactive protein, procalcitonin), use of vasopressor, duration of mechanical ventilation, anti-infective drugs, and other confounding risk factors that might be associated with adverse outcomes. Therefore, our results are still being validated in a prospective multicenter cohort enrolling non-critical patients.

## Conclusion

Critically ill diabetic patients with AKI and poor prognosis were more likely to have abnormal iron metabolism. Increased SF and decreased TRF are important risk factors for AKI and 6-month all-cause death in critical diabetic patients. The renal prognosis and survival rate of diabetic patients may be predicted by SF levels clinically.

## Data Availability Statement

The datasets presented in this study can be found in online repositories. The names of the repository/repositories and accession number(s) can be found in the article/supplementary material.

## Author Contributions

MM: study design, data analysis, and writing the manuscript. YG: study design, data analysis, data collection, and writing the manuscript. LD: study design and revision of the manuscript. YL: data collection and data analysis. NX and YL data collection and revision of the manuscript. All authors contributed to the article and approved the submitted version.

## Funding

This work was supported by the National Natural Science Foundation of China (no. 8176030057), the Guangxi Natural Science Foundation (no. 2018GXNSFBA050040), Guangxi Clinical Research Center for Urology and Nephrology (no. 2020AC03006), and the Scientific Research and Technological Development Program of Guangxi (no. GuiKeGong 1598011-6).

## Conflict of Interest

The authors declare that the research was conducted in the absence of any commercial or financial relationships that could be construed as a potential conflict of interest.

## Publisher’s Note

All claims expressed in this article are solely those of the authors and do not necessarily represent those of their affiliated organizations, or those of the publisher, the editors and the reviewers. Any product that may be evaluated in this article, or claim that may be made by its manufacturer, is not guaranteed or endorsed by the publisher.
